# Expression levels and clinical values of miR-92b-3p in breast cancer

**DOI:** 10.1186/s12957-021-02347-7

**Published:** 2021-08-11

**Authors:** Yu Du, Zhuang Miao, Kedi Wang, Yan Lv, Lijuan Qiu, Lusheng Guo

**Affiliations:** 1 Department of Laboratory, Beijing Friendship Hospital, Capital Medical University, Beijing, 100050 China; 2 Department of Laboratory, Affiliated Hospital of Jilin Medical College, No 81 HuaShan Road, Jilin, 132013 China; 3 Department of Laboratory, Beijing Public Security Hospital, Beijing, 100050 China; 4 Blood Transfusion Department, Beijing Children’s Hospital, Capital Medical University, Beijing, 100045 China

**Keywords:** miR-92b-3p, Breast cancer, Pathological features, Diagnosis, Prognosis

## Abstract

**Background:**

miR-92b is a carcinogenic miRNA that has great potential as a biomarker for disease prognosis, diagnosis, and treatment in the clinic. It is of great significance to analyse the relationship between miR-92b and the clinicopathological characteristics of cancer patients. This paper aimed to investigate the expression levels and clinical values of miR-92b-3p in breast cancer (BC).

**Methods:**

Altogether, 112 female BC patients who were treated in our hospital were included as a study group, and 108 healthy women who came to our hospital for physical examinations were included as a control group. miR-92b-3p expression in the serum of subjects in both groups was detected by fluorescence quantitative PCR (RT-PCR) to analyse the correlation of this miRNA with the patients’ pathological features and prognoses. The diagnostic value of miR-92b-3p expression for BC was analysed by plotting a receiver operating characteristic (ROC) curve.

**Results:**

miR-92b-3p expression was remarkably higher in the study group (*P* < 0.05), and its area under the curve (AUC) for detecting BC was 0.88. The expression was correlated with the tumour size, degree of differentiation, TNM staging, and lymphatic metastasis (*P* < 0.05). miR-92b-3p was significantly positively correlated with the TNM staging (*r* = 0.40, *P* < 0.05), was significantly negatively correlated with the degree of differentiation of the breast cancer cells (*r* =  − 0.35, *P* < 0.05), and was significantly positively correlated with the expression of carbohydrate antigen 125 (CA125) (*r* = 0.39, *P* < 0.05). The overall survival rate (OSR) of the 99 patients who had follow-up was 73.74%. The survival status was remarkably better in the low expression group (*P* < 0.05). miR-92b-3p expression was remarkably higher in the death group (*P* < 0.05). The AUC of miR-92b-3p alone in the death and survival groups was 0.76.

**Conclusion:**

miR-92b-3p expression obviously rises in the serum of BC patients and is closely related to the clinical staging, degree of differentiation, and CA125 in BC, so the detection of this miRNA is of great significance to the diagnosis and prognostic evaluation of BC. This miRNA can be used as a potential biomarker for the diagnosis and prognosis of the disease.

## Background

Cancers, one of the top ten malignant diseases, are a great threat to the life and safety of all humankind. Although their overall mortality rate continues to decline, their incidence in females has slightly risen [1]. Worldwide, approximately 1.2 million women are newly confirmed to have breast cancer (BC), and approximately 500,000 patients die from the disease every year [2]. In 2015, 40,290 American women died from BC, and approximately 200,000 were confirmed to have invasive BC, while in 2016, approximately 3,560,000 women suffered from BC [3]. In China, although the incidence and mortality rate of the disease are relatively low, it is still one of the most common malignant tumours threatening women’s health, and the incident and mortality rate rise to first and sixth places, respectively, among all female cancers [4]. In 2014, approximately 278,900 cases were seen of newly developed BC in China, accounting for 16.51% of the new female cases, while approximately 66,000 patients have died from the disease in this country, accounting for 7.82% of women’s deaths related to cancers [5]. The age-specific incidence of BC rises significantly in those patients older than 20 years old, and the disease is often seen in patients aged 55–60 years old [6].

As a kind of noncoding RNA (approximately 22 nt long) that is found in many organisms, such as animals, plants, and viruses, microRNAs (miRNAs) exert a pivotal function in readjusting gene expression, so their identification and annotation have become a major focus of epigenomic research in the past decade [7]. According to some studies, miRNAs are involved in the process of tumour occurrence and development as proto-oncogenes or tumour suppressor genes [8]. Given their great potential to become biomarkers for the prognosis, diagnosis, and treatment of diseases in clinical practice, it is of great significance to analyse the relationship between miRNAs and the clinicopathological characteristics of tumour patients [9]. miR-92b is a carcinogenic miRNA, and miR-92b-3p has a potential role in the occurrence and metastasis of colorectal cancer [10]. In addition, the latter inhibits pancreatic cancer by regulating Gabra3 expression and is related to patient prognosis.

Therefore, miR-92b-3p expression in both groups was detected, and its correlation with BC patients’ pathological features and prognoses was analysed to evaluate the clinical significance of the expression in the patients.

## Methods

### General information

Altogether, 112 female BC patients who came to our hospital for treatment were included as the study group, with an average age of 50.64 ± 4.58 years. In addition, 108 healthy women who came to our hospital for physical examinations were included as the control group, and their average age was 50.49 ± 5.21 years. This study was approved by the Ethics Committee of our hospital. The patients and their family members were informed of the experimental process and then signed the informed consent form.

### Inclusion and exclusion criteria

Inclusion criteria are as follows:

1. Patients were accompanied by their family members on admission.
2. Patients had complete clinicopathological data.
3. Patients were confirmed to have BC by postoperative pathology.
4. Patients did not have preoperative treatment with radiotherapy and chemotherapy.

Exclusion criteria are as follows:

1. Those patients who had a previous history of mental illness or a family history of psychosis.
2. Those patients with a history of autoimmune deficiencies, severe organ diseases, or drug dependence.
3. Those patients who could not cooperate during the examinations due to aphasia, dysphoria, unconsciousness, or communication disorders.

### Methods

After 12 h of fasting, 10 mL of venous blood was collected from the subjects in both groups and then put into anticoagulation tubes for 60 min of coagulation (20–25 °C). After centrifugation (Sichuan Shuke Instrument Co., Ltd., TG112) at 1369.55xp (4 °C, 15 min), the blood was separated to obtain the upper serum, which was stored in a freezer (− 70 °C). Fluorescence quantitative PCR (RT-PCR) was performed to detect miR-92b-3p expression. First, total RNA was extracted from the serum based on the instructions of the TRIzol kits (Shenyang Wanlei Biotechnology Co., Ltd., WLA088b). To eliminate any contamination with genomic DNA, the template RNA was digested with DNase1 (RNA free) (Shanghai Hengfei Biotechnology Co., Ltd., K003399P). An ultraviolet spectrophotometer (Shanghai Hengfei Biotechnology Co., Ltd., UV-1100) was used to determine the purity and concentration of the RNA. After the RNA concentration was adjusted to 500 ng/μL, the RNA samples were reverse transcribed into cDNA, and the steps were strictly conducted based on the instructions of the cDNA reverse transcription kits (Shanghai EvenBridge Biotechnology Co., Ltd., 4,368,814). The RT-PCR system (20 μL) was the 2 × Ultra SYBR one step with Qrt-PCR Buffer (10 μL), RNA template (2 μL), nuclear-free water (5.5 μL), upstream and downstream primers (1 μL each), and the Super enzyme mix (0.5 μL). Conditions were predenaturation (95 °C, 10 min), denaturation (95 °C, 15 s), and annealing and extension (60 °C, 1 min), which were cycled for 40 times. Primers of this experiment were designed by the Primer Premier 5.0 (Seebio, P7359) primer design software and generated by Tianjin Saier Biotechnology, Inc. U6 was the internal reference for miR-92b-3p. See Table 1 for the primer sequences. During the process of amplification with the cycle number (Ct) value and the fluorescence signals from the background, we began to enter the number of cycles corresponding to the inflexion point of the exponential growth phase. Relative miR-92b-3p expression was calculated by 2^−△Ct^. Through an enzyme-linked immunosorbent assay (ELISA), the carbohydrate antigen 125 (CA125; human CA125 ELISA kits were purchased from Beijing Biolab Science And Technology Co., Ltd., ZN2049-JPT) was measured, with the steps conducted in strict accordance with the kit instructions.

**Table 1:** Primer sequences

Primers	Positive primers	Reverse primers
miR-92b-3p	ACACTCCAGCTGGGTATTGCACTCGTCCCGGC	TCTCAACTGGTGTCGTGGAGTCGGCAATTCAGT
U6	CTCGCTTCGGCAGCACA	AACGCTTCACGAATTTGCGT

### Outcome measures

The miR-92b-3p and CA125 expression in the serum of subjects in both groups was detected. The correlation of this miRNA with the patients’ pathological features was analysed. The correlation with the TNM staging, degree of differentiation, and CA125 in BC was also analysed. At the 6th, 12th, 24th, 36th, 48th, and 60th months after treatment, the patients were followed-up by telephone or outpatient reexaminations for 5 years to record their survival status and analyse the correlation of the survival rate with the miR-92b-3p expression. A receiver operating characteristic (ROC) curve was plotted to analyse the values of miR-92b-3p alone for the diagnosis and prognostic evaluation of BC.

### Statistical methods

In this study, SPSS 20.0 (IBM Corp, Armonk, NY, USA) was used for the statistical analysis. Figures were plotted using GraphPad Prism 7 (GraphPad Software, Inc., San Diego CA, USA). [*n* (%)] was used to express the categorical data, which were compared between the groups by the chi-square test ($\overline{\mathrm{x}}\pm \mathrm{s }$) was used to express the continuous data, which were compared between the two groups by the *t*-test. The Spearman correlation coefficient was used for the correlation analysis, and an ROC curve was plotted to evaluate the sensitivity and specificity of miR-92b-3p alone. When *P* < 0.05, the difference was considered statistically significant.

## Results

### Comparison of general information

We collected the basic data of participants in both groups, including sex, body mass index (BMI), and smoking and drinking history. The analysis results of the *χ*^2^ test and the independent samples *t*-test showed that the demographic information was not significantly different between the study and control groups (*P* > 0.05). See Table 2.

**Table 2:** Comparison of demographic information ($\overline{\mathrm{x}}\pm \mathrm{sd }$)/n [%]

	Study group (*n* = 112)	Control group (*n* = 108)	*t*/*X*^2^	*P*
Age (years)	50.64 ± 4.58	50.49 ± 5.21	0.23	0.82
BMI (kg/m^2^)	22.53 ± 2.24	22.49 ± 2.26	0.13	0.90
Smoking or not			0.16	0.69
Yes	21 (18.75)	18 (16.67)	-	-
No	91 (81.25)	90 (83.33)	-	-
Drinking or not			0.10	0.75
Yes	29 (25.89)	30 (27.78)	-	-
No	83 (74.11)	78 (72.22)	-	-
Place of residence			0.10	0.75
City	63 (56.25)	63 (58.33)	-	-
Countryside	49 (43.75)	45 (41.67)	-	-
TNM staging			-	-
I-II	55 (49.11)	0 (0.00)	-	-
III	57 (50.89)	0 (0.00)	-	-

### Comparison of miR-92b-3p expression

**Fig. 1:**
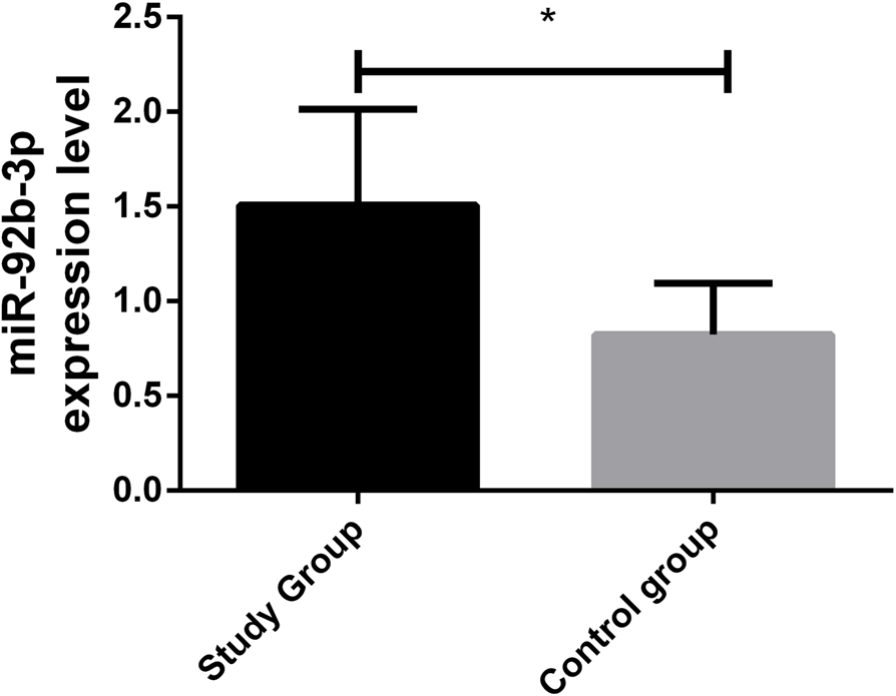
Comparison of miR-92b-3p expression. The expression of miR-92b-3p in the study group was remarkably higher than that in the control group. Asterisk indicates *P* < 0.05 for the comparison between two groups

The qRT-PCR results showed that the expression of miR-92b-3p in the study group was significantly higher than that in the control group (*P* < 0.05). See Fig. 1.

### Diagnostic value of single miR-92b-3p for BC

**Fig. 2:**
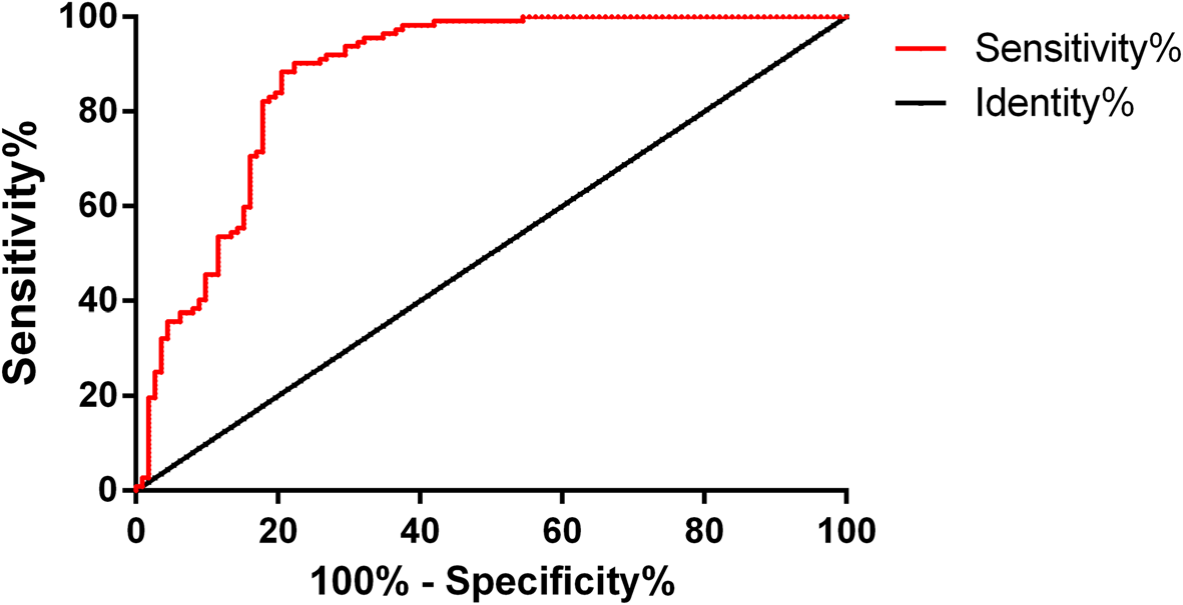
ROC curve of single miR-92b-3p. The AUC of single miR-92b-3p was 0.88

The ROC curve was used to analyse the diagnostic value of miR-92B-3p in BC (Table 3). Based on the area under the curve (AUC) of miR-92b-3p alone, its diagnostic value for BC could be judged. AUC is an evaluation index to measure the pros and cons of a two-class model, which indicates the probability that the predicted positive example is ranked in front of the negative example. The AUC, specificity and sensitivity of miR-92B-3p for the diagnosis of BC were 0.88, 79.46%, and 88.39%, respectively. See Table 4 and Fig. 2.

**Table 3:** Value of miR-92b-3p alone for predicting the prognosis of BC

	Specificity	Sensitivity	Optimal cut-off value	Youden index	AUC	*P*	95% confidence interval	
							Lower	Upper
miR-92b-3p	73.08%	75.34%	< 1.65	0.06	0.76	0.00	0.65	0.88

**Table 4:** Diagnostic value of single miR-92b-3p for BC

	Specificity	Sensitivity	Optimal cut-off value	Youden index	AUC	*P*	95% confidence interval	
							Lower	Upper
miR-92b-3p	79.46%	88.39%	> 0.32	0.02	0.88	0.00	0.83	0.92

### Correlation of miR-92b-3p expression with pathological features

The correlation of miR-92b-3p expression with BC patients’ pathological features was analysed (Table 5). The univariate analysis showed that the expression of miR-92b-3p had no significant correlation with age or menopausal status (*P* > 0.05) but was correlated with the tumour size, the degree of differentiation, the TNM staging, and lymphatic metastasis (*P* < 0.05). The expression level of miR-92b-3p was higher in patients with larger tumours, tumours with a lower differentiation, a higher TNM stage, and lymph node metastasis (*P* < 0.05).

**Table 5:** Correlation of miR-92b-3p expression with clinicopathological features ($\overline{\mathrm{x}}\pm \mathrm{s }$)

	Number of cases	miR-92b-3p	t	*P*
Age (years)			0.48	0.63
< 50	52	1.56 ± 0.35		
≥ 50	60	1.59 ± 0.31		
Tumour size (cm)			6.21	0.00
< 2	43	1.37 ± 0.32		
≥ 2	69	1.72 ± 0.27		
Degree of differentiation			5.88	0.00
Moderately + highly differentiated	44	1.38 ± 0.34		
Lowly differentiated	68	1.74 ± 0.30		
TNM staging			6.47	0.00
I-II	55	1.41 ± 0.42		
III	57	1.86 ± 0.31		
Menopause			0.89	0.37
Yes	41	1.55 ± 0.38		
No	71	1.62 ± 0.41		
Lymphatic metastasis			4.63	0.00
No	40	1.40 ± 0.37		
Yes	72	1.75 ± 0.39		

### Correlation of miR-92b-3p expression with TNM staging and degree of differentiation

**Fig. 3:**
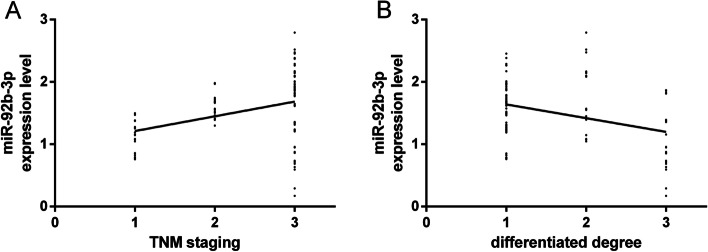
Correlation of miR-92b-3p expression with TNM staging and degree of differentiation of BC. **A** miR-92b-3p expression was positively correlated with the TNM staging of BC. **B** miR-92b-3p expression was negatively correlated with the degree of differentiation of BC. In **B**, 1 indicates a low differentiation, 2 indicates a moderate differentiation, and 3 indicates a high differentiation

The correlation of miR-92b-3p expression with the TNM staging and degree of differentiation of BC was analysed (Fig. 3). A Spearman correlation analysis revealed that miR-92b-3p was significantly and positively correlated with the TNM staging (*r* = 0.40, *P* < 0.05) and significantly and negatively correlated with the degree of differentiation of the BC cells (*r* =  − 0.35, *P* < 0.05).

### Correlation of miR-92b-3p with CA125

**Fig. 4:**
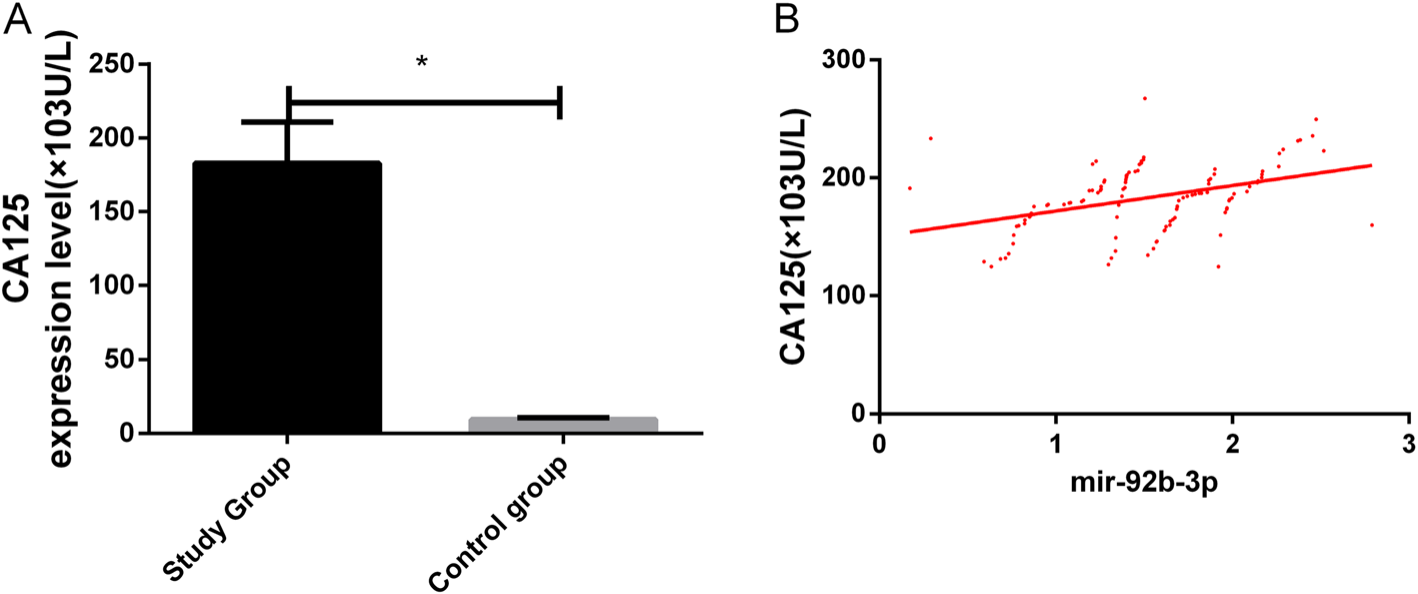
Correlation of miR-92b-3p with CA125. **A** CA125 expression in the study group was remarkably higher than that in the control group. **B** miR-92b-3p and CA125 expression was positively correlated in BC patients. Asterisk indicates *P* < 0.05 for the comparison between two groups

The correlation of miR-92b-3p with CA125 was analysed. CA125 expression was remarkably higher in the study group (*P* < 0.05) and positively correlated with miR-92b-3p expression (*r* = 0.39, *P* < 0.05) (Fig. 4).

### Correlation of survival status with miR-92b-3p expression

**Fig. 5:**
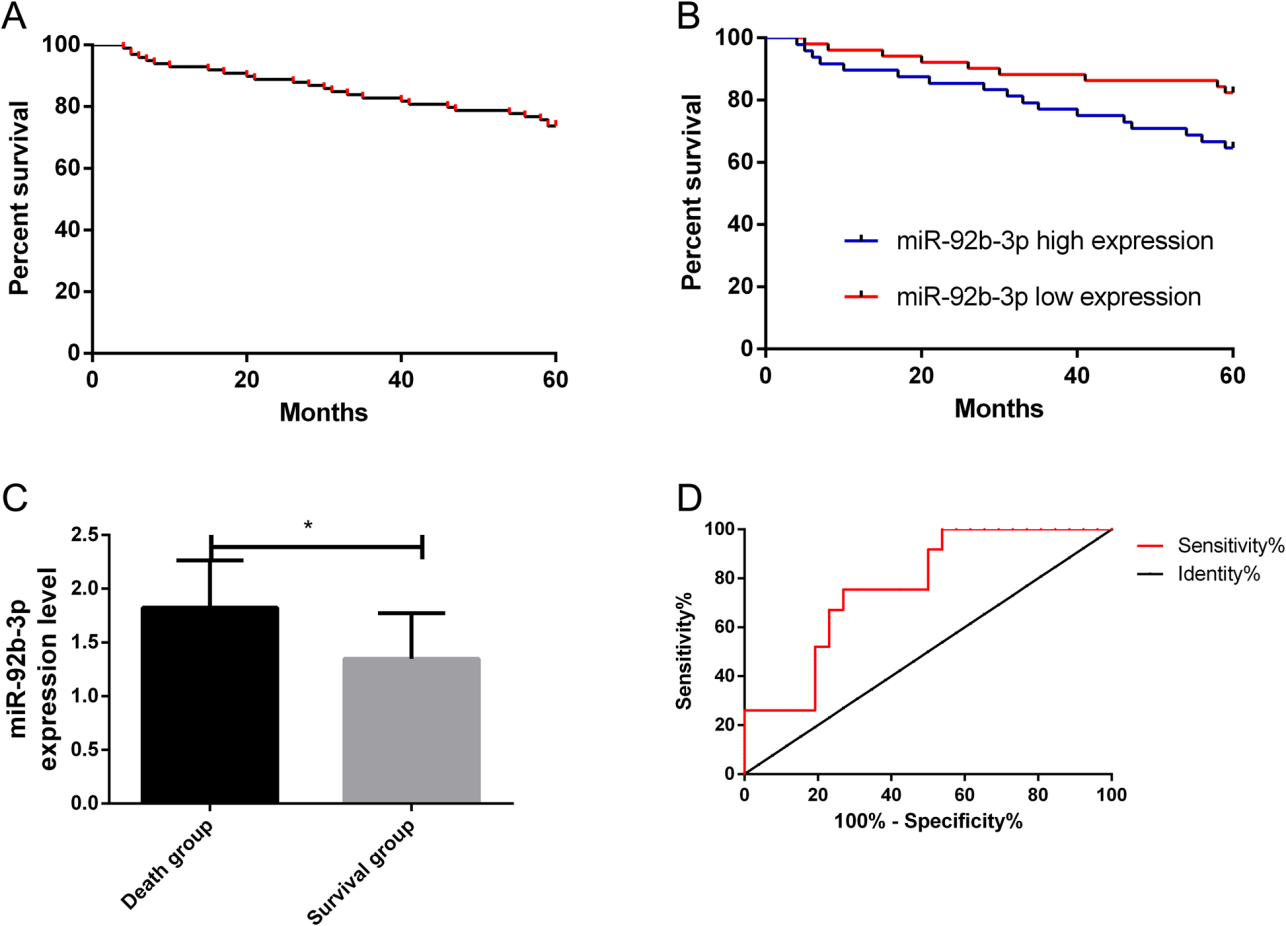
Correlation of survival status with miR-92b-3p expression. **A** The OSR of the 99 interviewed BC patients was 73.74%. **B** The OSR in the low expression group was remarkably higher than that in the high expression group. **C** miR-92b-3p expression in the death group was remarkably higher than that in the survival group. **D** The AUC of miR-92b-3p alone in the death and survival groups was 0.76. Asterisk indicates *P* < 0.05 when there is a comparison between two groups

During the 5-year follow-up, 99 patients were successfully followed-up, while 13 patients were lost to follow-up. Based on the median miR-92b-3p expression, 99 respondents were grouped into two groups: the high expression group (48 cases) and the low expression group (51 cases). According to the survival status, the patients were divided into the survival group (73 patients) and the death group (26 patients). The OSR of the followed-up patients was 73.74%. Compared with the patients with a high expression of miR-92b-3p, the survival rate was remarkably higher in the low expression group (*P* < 0.05). The miR-92b-3p expression was remarkably higher in the death group (*P* < 0.05). ROC analysis showed that the AUC of predicting patient survival by miR-92b-3p was 0.76. This suggests that miR-92b-3p can predict the prognosis and survival of patients (Fig. 5).

## Discussion

BC patients usually have a poor prognosis because of the lack of an early non-invasive diagnosis of the disease and due to the development of drug resistance and metastasis during treatment [11]. In 2020, the mortality rate of BC is predicted to be 13.4/100,000 in the EU, among which the rate will rise and is predicted to be 15.3/100,000 in Poland [12]. Therefore, it is worth seeking satisfactory molecular markers for the early diagnosis and treatment of the disease [13]. miRNAs have a significant effect on the early diagnosis and prognostic evaluation of BC and on reversing the drug resistance of cancers [14]. All kinds of miRNAs have abnormal expression in BC [15, 16], and these maladjusted miRNAs play an antitumour or carcinogenic role in the disease and are involved in its pathological process [17, 18]. There is abnormal expression of miR-92b-3p in colorectal cancer [19, 20] and oesophageal squamous cell carcinoma [21]. Therefore, we analysed the expression and clinical significance of this miRNA in BC.

According to Uotani et al., the serum miR-92b-3p expression is remarkably higher in patients with synovial sarcomas than in healthy individuals [22], and this miRNA is upregulated in cervical cancer [23]. In this study, miR-92b-3p expression was remarkably higher in the study group. This indicates that this miRNA is specifically expressed in BC and that it plays a possible carcinogenic role in the disease. In the validation cohort, six miRNA signals, including miR-92b-3p, were added to four clinicopathological factors, and their AUC for predicting treatment responses remarkably increased from 0.79 to 0.90 compared with patients with stable diseases [24]. In our study, the AUC of single miR-92b-3p was 0.88, which suggests that the single detection of this miRNA has a relatively high diagnostic value for BC. miR-92b-3p expression was not obviously related to age or menopause but was correlated with the tumour size, the degree of differentiation, the TNM staging, and lymphatic metastasis. This reveals that miR-92b-3p has a significant effect on the occurrence and development of BC. According to some studies, APE1 enhances the processing of miR-92b-3p, thus inhibiting LDLR expression and promoting the progression of cervical cancer [25]. According to independent verifications, the detection of this miRNA can identify patients with metastatic colorectal cancer who would benefit from the combined treatment of bevacizumab and oxaliplatin-based chemotherapy [26]. In prostate cancer, the expression of miR-92b-3p is also upregulated, which is closely related to the presence of distant metastases, tumour lymph node metastasis and poor prognosis in patients with prostate cancer [27]. In this study, miR-92b-3p expression was significantly and positively correlated with the TNM staging of BC but was significantly and negatively correlated with the degree of differentiation of the BC cells. This suggests that this miRNA can be used to judge the severity of BC and may be a potential therapeutic target for patients, which is significant for the prognostic evaluation of the disease. CA125 expression in the two groups was also detected. BC is the most common hormone-related gynaecologic cancer, and CA125 is an important biomarker for an early detection and the monitoring of its pathogenesis [28]. In this study, CA125 expression was remarkably higher in the study group and positively correlated with miR-92b-3p in BC patients. Additionally, CA125 is a serum marker for BC [29], so this miRNA may be a potential biomarker for the disease and has a certain value for its early diagnosis. In this study, 99 patients were followed-up for 5 years, and 13 were lost, with a return visit rate of 88.39% and a 5-year OSR of 73.74%. The survival status was remarkably better in the low expression group, and miR-92b-3p expression was remarkably higher in the death group. This demonstrates that high miR-92b-3p expression indicates a poor prognosis for BC patients. The AUC of single miR-92b-3p for predicting prognosis was 0.76, indicating that the single detection of this miRNA has a relatively high prognostic value for BC. This demonstrates that clinical therapeutic schemes for BC patients can be adjusted and improved by detecting miR-92b-3p expression, thus improving the patients’ prognosis and survival rate.

In this study, miR-92b-3p expression in both groups was detected to analyse its correlation with BC patients’ pathological features and prognoses as well as its clinical significance in patients with BC to provide theoretical bases for diagnosing and treating BC early. However, there are still limitations to this study. In vitro and in vivo experiments should be further performed to verify the specific role of miR-92b-3p in BC. The mechanisms by which this miRNA is differentially expressed are still unclear. We hope that the research in BC will be improved in future studies to provide a more scientific reference for the clinical treatment of BC. At present, some studies have explored the biological effects of miR-92b-3p on BC cells and the potential mechanisms of action. Liu et al. [30] found that the expression of miR-92b was downregulated in BC cells and could promote starvation and rapamycin-induced autophagy by targeting enhancer of zeste homologue 2 (EZH2). In addition, Li et al. [31] found that miR-92b can directly target the 3′-UTR of Gabra 3 mRNA to suppress the migration and invasion of triple-negative breast cancer. These studies demonstrate the antitumour effect of miR-92b in BC at the cellular level, which is somewhat inconsistent with our results. Other evidence has revealed that miR-92b plays a tumour-promoting role in some other tumours. For example, in colorectal cancer, the upregulation of small nucleolar RNA host gene 14 (SNHG 14) suppresses the progression and metastasis of colorectal cancer cells by competing with miR-92b-3p [20]. This shows that different transcriptional results may affect the role of miR-92b, which needs to be further verified in future studies.

## Conclusion

In summary, miR-92b-3p expression obviously rises in the serum of BC patients and is closely related to the clinical staging, the degree of differentiation, and CA125 in BC, so the detection of this miRNA is of great significance to the diagnosis and prognostic evaluation in BC patients.

## Data Availability

The datasets used and/or analysed during the current study are available from the corresponding author on reasonable request.
